# Identification and characterization of N6‐methyladenosine modification of circRNAs in glioblastoma

**DOI:** 10.1111/jcmm.16750

**Published:** 2021-06-27

**Authors:** Yuhao Zhang, Xiuchao Geng, Jianglong Xu, Qiang Li, Liangchao Hao, Zhaomu Zeng, Menglin Xiao, Jia Song, Fulin Liu, Chuan Fang, Hong Wang

**Affiliations:** ^1^ Department of Neurosurgery Affiliated Hospital of Hebei University Baoding China; ^2^ School of Clinical Medicine Hebei University Baoding China; ^3^ School of Medicine Taizhou University Taizhou China; ^4^ Faculty of Integrated Traditional Chinese and Western Medicine Hebei University of Chinese Medicine Shijiazhuang China; ^5^ Faculty of Acupuncture‐Moxibustion and Tuina Hebei University of Chinese Medicine Shijiazhuang China; ^6^ Department of Plastic Surgery Shaoxing People's Hospital Shaoxing China; ^7^ School of Basic Medicine Hebei University Baoding China; ^8^ Office of Academic Research Affiliated Hospital of Hebei University Baoding China; ^9^ Hebei Key Laboratory of Chinese Medicine Research on Cardio‐cerebrovascular Disease Hebei University of Chinese Medicine Shijiazhuang China

**Keywords:** CircRNA, decoration pattern, GBM, glioblastoma, N6‐methyladenosine modification, ncRNA, noncoding RNA

## Abstract

This research systematically profiled the global N6‐methyladenosine modification pattern of circular RNAs (circRNAs) in glioblastoma (GBM). Based on RNA methylation sequencing (MeRIP sequencing or N6‐methyladenosine sequencing) and RNA sequencing, we described the N6‐methyladenosine modification status and gene expression of circRNAs in GBM and normal brain tissues. N6‐methyladenosine–related circRNAs were immunoprecipitated and validated by real‐time quantitative PCR. Bioinformatics analysis and related screening were carried out. Compared with those of the NC group, the circRNAs from GBM exhibited 1370 new N6‐methyladenosine peaks and 1322 missing N6‐methyladenosine peaks. Among the loci associated with altered N6‐methyladenosine peaks, 1298 were up‐regulated and 1905 were down‐regulated. The N6‐methyladenosine level tended to be positively correlated with circRNA expression. Bioinformatics analysis was used to predict the biological function of N6‐methyladenosine–modified circRNAs and the corresponding signalling pathways. In addition, through PCR validation combined with clinical data mining, we identified five molecules of interest (BUB1, C1S, DTHD1, F13A1 and NDC80) that could be initial candidates for further study of the function and mechanism of N6‐methyladenosine–mediated GBM development. In conclusion, our findings demonstrated the N6‐methyladenosine modification pattern of circRNAs in human GBM, revealing the possible roles of N6‐methyladenosine–mediated novel noncoding RNAs in the origin and progression of GBM.

## 1 INTRODUCTION

Glioma is a common brain tumour that accounts for nearly 80% of all primary brain neoplasms. Among them, glioblastoma (GBM) is a life‐threatening tumour with a worse survival outcome. Despite the use of multiple aggressive treatments, such as surgery and/or chemoradiotherapy, the survival rate of GBM patients is relatively low.^1^, ^2^, ^3^ Therefore, to develop a better treatment strategy, it is important to understand the molecular features of GBM occurrence. Recent epigenetic studies have found that RNA posttranscriptional modification plays an essential role in regulating cell growth and metabolism as well as the biological behaviour of tumours. Over 60% of RNA modifications belong to N6‐methyladenosine, which is also a prevalent epigenetic modification in eukaryotic mRNA. The modification process is reversible and is completed by ‘writers’, ‘erasers’ and ‘readers’.^4^, ^5^, ^6^

Interestingly, in addition to mRNAs, the well‐known noncoding RNAs—circRNAs (a class of covalently linked single‐stranded closed circRNAs without a 3′‐end poly(A) tail or 5′‐end cap)—also possess extensive N6‐methyladenosine modification sites.^7^ CircRNAs are closely related to the occurrence and development of glioma.^8^, ^9^ Moreover, a previous study suggested that different sets of principles can affect N6‐methyladenosine biogenesis in circRNAs and mRNA because a number of N6‐methyladenosine‐circRNAs are produced from exons whose corresponding mRNAs do not contain N6‐methyladenosine peaks. Despite this, there is a lack of studies on N6‐methyladenosine modification of circRNAs, and the role of N6‐methyladenosine modification of circRNAs in GBM pathogenesis remains unclarified.^10^, ^11^, ^12^

In this study, we reported for the first time the circRNA‐based analysis of N6‐methyladenosine modification in GBM tissue and normal brain tissue, which proved that there was a high degree of difference and diversity in the N6‐methyladenosine modification patterns between GBM and control groups. Meanwhile, abnormal N6‐methyladenosine modification of circRNA in GBM has been shown to be involved in transcriptional regulation and cancer‐associated pathways. We hope that this study will help further investigate the potential effects of N6‐methyladenosine modification on GBM pathogenesis.

## 2 MATERIALS AND METHODS

### 2.1 Patients & samples

Glioma tissues (confirmed as GBM by postoperative pathological diagnosis) and normal cerebral cortex tissues (traumatic brain contusion and laceration combined with cerebral hernia, requiring internal decompression) were collected intraoperatively. After the samples were isolated, they were rinsed with normal saline and immediately transferred to a 1.8‐ml RNA‐free cryopreserved tube, which was stored in a refrigerator at −80°C for RNA isolation. Five clinical samples from the GBM group and normal control group (NC group) were selected for N6‐methyladenosine sequencing and RNA sequencing, and the remaining samples were stored for verification (Table S1). Ethical approval for this study was obtained from the Ethics Committee of the Affiliated Hospital of Hebei University, and written informed consent was received from all participants.

### 2.2 Preparation and sequencing of MeRIP and RNA libraries

N6‐methyladenosine sequencing and RNA sequencing services were provided by CloudSeq Biotech Inc (Shanghai, China). Briefly, the GENSeqTM N6‐methyladenosine RNA IP Kit (GenSeq Inc, China) was used to perform N6‐methyladenosine RNA immunoprecipitation according to the manufacturer's protocol. Both N6‐methyladenosine IP and input (without immunoprecipitation) samples were subjected to RNA sequencing library construction using a NEBNext^®^ Ultra II Directional RNA Library Prep Kit (New England Biolabs). The RNA library construction process was similar. The library quality was assessed using a Bioanalyser 2100 system (Agilent Technologies, CA, USA). The NovaSeq 6000 system (150‐bp paired‐end reads; Illumina) was employed to perform library sequencing.

### 2.3 Bioinformatics analysis

The paired‐end sequences were acquired from an Illumina NovaSeq 6000 sequencer and subjected to quality control (Q30). Subsequently, 3′ adapter trimming and low‐quality sequence removal were performed by cutadapt 1.9.3 software.^13^ The alignment of clean sequences was performed against a reference genome (hg19, UCSC) using STAR software.^14^ The identification of circRNAs was carried out by DCC software based on the aligned sequences.^15^ Data normalization and differential circRNA expression analysis were performed using EdgeR software (v3.16.5),^16^ and the high‐quality sequences from all libraries were aligned against the reference genome using HiSat2 software (v2.0.4).^17^ MACS software was used to screen potential methylated sites on RNAs (peaks),^18^ whereas diffReps was used to identify differentially methylated sites.^19^ The peaks found by these two programmes as overlapping exons of mRNA and circRNA were identified and extracted by homemade scripts. Gene ontology (GO) enrichment and Kyoto Encyclopedia of Genes and Genomes (KEGG) pathway analyses were utilized to determine the source genes of differentially methylated circRNAs and differentially expressed circRNAs.

### 2.4 Gene‐specific N6‐methyladenosine qPCR validation

Five genes with differentially methylated sites according to N6‐methyladenosine sequencing and RNA sequencing were subjected to reverse transcription (RT)‐qPCR. A portion of the fragmented RNA was saved for use as the input control. The remaining RNA was bound to anti‐N6‐methyladenosine antibody‐coupled beads, and the N6‐methyladenosine‐containing RNA was then immunoprecipitated and eluted from the beads. Both the N6‐methyladenosine‐IP sample and input control were subjected to RT‐qPCR with gene‐specific primers. The primer sequences are listed in Table S2.

### 2.5 Statistical analysis

The mean ±standard deviation (SD) was calculated from the data of 3 independent experiments. Statistical tests were conducted using SPSS 25.0 and GraphPad Prism 7.0 software. Paired Student's *t* tests were performed between GBM group and NC group samples. One‐way ANOVA was utilized to compare the differences among three or more groups. Data were considered statistically significant as follows: *P‐value <.05, **P‐value <.01, ***P‐value <.001 and ****P‐value <.0001. All experiments were repeated three times independently.

## 3 RESULTS

### 3.1 Overall N6‐methyladenosine modification pattern in mRNAs from both groups

**FIGURE 1:**
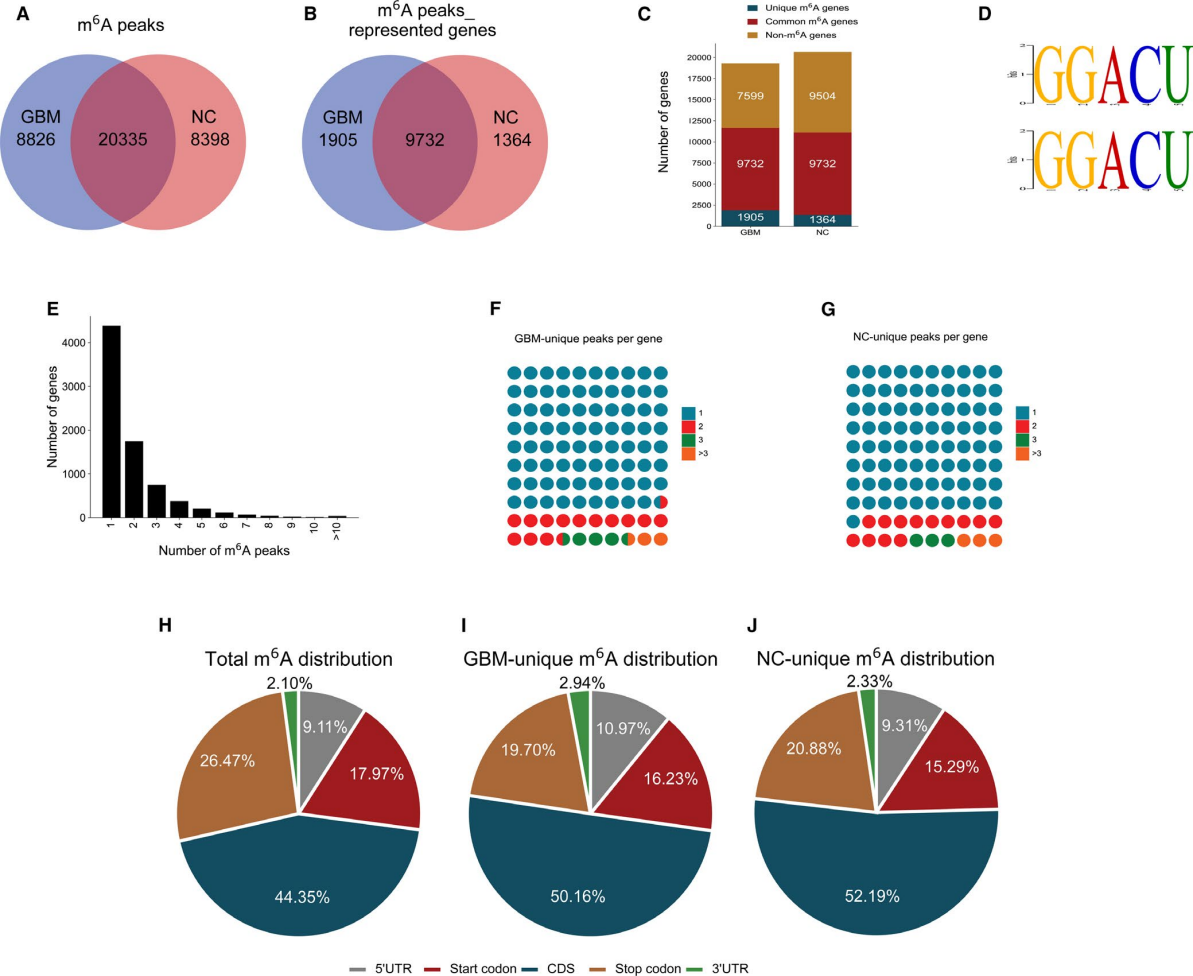
Transcriptome‐wide N6‐methyladenosine sequencing and determination of N6‐methyladenosine peaks. (A) Venn diagram of all N6‐methyladenosine peaks in the two groups; (B) venn diagram of N6‐methyladenosine peak‐represented genes in the two groups; (C) the amounts of GBM‐unique, NC‐unique and common N6‐methyladenosine genes; (D) top N6‐methyladenosine motifs enriched from all identified N6‐methyladenosine peaks; (E) the distribution of N6‐methyladenosine–modified peaks in each gene; (F) the distribution of N6‐methyladenosine–modified peaks per gene in GBM‐unique N6‐methyladenosine genes; (G) the distribution of N6‐methyladenosine–modified peaks per gene in NC‐unique N6‐methyladenosine genes; (H‐J) the proportions of N6‐methyladenosine peaks in the study regions in all samples (H); the proportions of unique N6‐methyladenosine peak distribution in the study regions in GBM samples (I); the proportions of unique N6‐methyladenosine peak distribution in the study regions in the NC samples (J). GBM: Glioblastoma group; NC: Normal control group

Human GBM tissue and normal brain tissue (n = 5) were selected for RNA sequencing and transcriptome‐wide N6‐methyladenosine sequencing assays. A total of 29 161 N6‐methyladenosine peaks were identified by MACS in the GBM group, representing transcripts of 11 637 genes. In the NC group, 28 733 N6‐methyladenosine peaks were identified, which corresponded to 11 096 gene transcripts (Figure 1A,B). Among them, 20 335 individual N6‐methyladenosine peaks in 9732 N6‐methyladenosine–modified genes were detected in the two groups. Notably, the GBM group had 8826 new peaks and 8398 missing peaks compared to the NC group, revealing that the global N6‐methyladenosine modification patterns were markedly different between the GBM and NC groups (Figure 1A‐C).

The N6‐methyladenosine methylomes were further mapped by HOMER software. The top consensus motif in the 37,559 identified N6‐methyladenosine peaks was GGACU (Figure 1D). By analysing the N6‐methyladenosine–modified peaks of each gene, we found that 57% of all modified genes (4384/7757) had a unique N6‐methyladenosine modification peak. The majority of genes (6877/7757) had one to three N6‐methyladenosine–modified sites (Figure 1E). In particular, genes with GBM‐unique N6‐methyladenosine tended to have more N6‐methyladenosine–modified sites than genes with NC‐unique N6‐methyladenosine (genes with two N6‐methyladenosine–modified sites: 14.00% vs 13.00%; genes with three or more N6‐methyladenosine–modified sites: 6.50% vs 6.00%; Figure 1F,G).

Then, we analysed the distribution of N6‐methyladenosine in the whole transcriptome of GBM and NC samples. Both total and unique N6‐methyladenosine peaks from the two groups were analysed. N6‐methyladenosine peaks were categorized according to their locations in RNA transcripts as transcription start codons, 5′UTRs, coding sequences (CDSs), 3′UTRs and stop codons. In general, the N6‐methyladenosine peaks were especially enriched in the regions of the start codon (17.97%), CDS (44.35%) and stop codon (26.47%) (Figure 1H), which was consistent with previous N6‐methyladenosine–sequencing results. The GBM‐unique N6‐methyladenosine peaks showed a distinct pattern from NC‐unique peaks, with a relative decrease in N6‐methyladenosine modifications in the CDS region and stop codon region (Figure 1I,J). These 8826 GBM‐unique peaks included 968 peaks from the 5′UTR, 1432 from the start codon, 4427 from the CDS, 1739 from the stop codon and 260 from the 3′UTR. In general, N6‐methyladenosine peaks tend to occur in CDS regions, which means that N6‐methyladenosine modification is likely to play a crucial role in regulating encoded proteins, but the mechanism needs further study.

### 3.2 N6‐methyladenosine modification pattern in circRNAs from both groups

#### 3.2.1 Introduction to basic retouching patterns

**FIGURE 2:**
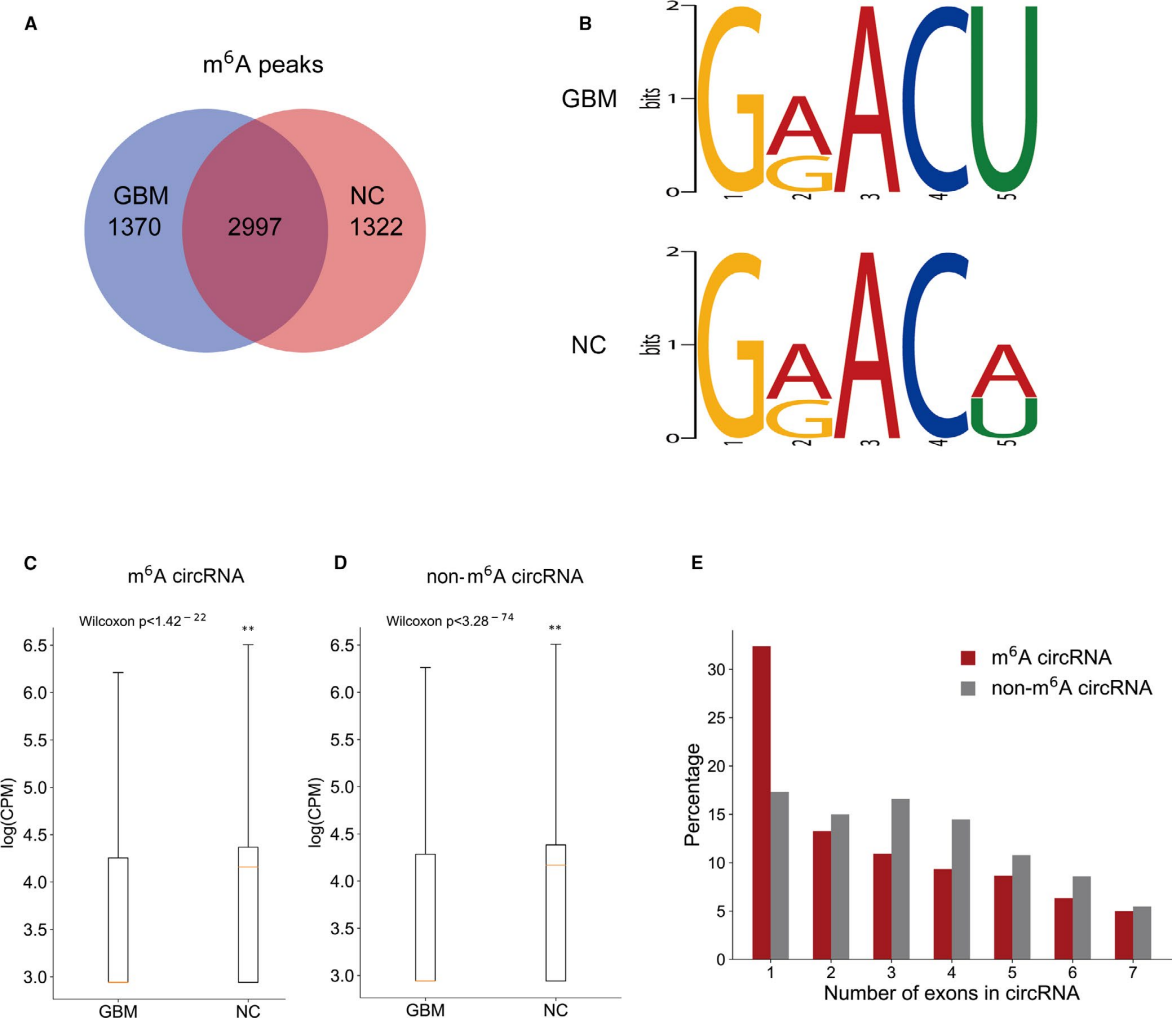
Differences in the expression of N6‐methyladenosine in circRNAs between the control and GBM groups. (A) Overlapping N6‐methyladenosine peaks in the circRNAs of the two groups; (B) the enriched motifs among the modified N6‐methyladenosine‐circRNAs of the two groups; (C, D) differential expression of N6‐methyladenosine‐circRNAs and non‐N6‐methyladenosine‐circRNAs in the two groups; (E) difference in the exon numbers of N6‐methyladenosine‐circRNAs and non‐N6‐methyladenosine‐circRNAs

Genome‐wide maps of N6‐methyladenosine‐modified circRNAs in the GBM and NC groups (n = 5 per group) were constructed. A total of 2,997 N6‐methyladenosine‐circRNA peaks overlapped in the control and GBM groups, whereas 1,322 N6‐methyladenosine‐circRNA peaks were only found in the control group but not in the GBM group, and 1,370 N6‐methyladenosine‐circRNA peaks were only detected in the GBM group but not in the control group (Figure 2A). Based on the motif analysis of 2000 circRNA peaks with the top enrichment score (−log10, P), a consensus sequence (GGACU) was identified in both the control and GBM groups (Figure 2B), suggesting that the data are reproducible. As demonstrated in Figure 2C,D, N6‐methyladenosine circRNA expression was relatively lower in the GBM group than in the control group, which was also true for non‐N6‐methyladenosine circRNAs, indicating that the downregulation of circRNAs in the GBM group seems to be unrelated to the assembly of N6‐methyladenosine. Most N6‐methyladenosine‐circRNAs and non‐N6‐methyladenosine‐circRNAs were frequently composed of a single one or more exons (Figure 2E).

#### 3.2.2 Distribution characteristics of N6‐methyladenosine modification sites

**FIGURE 3:**
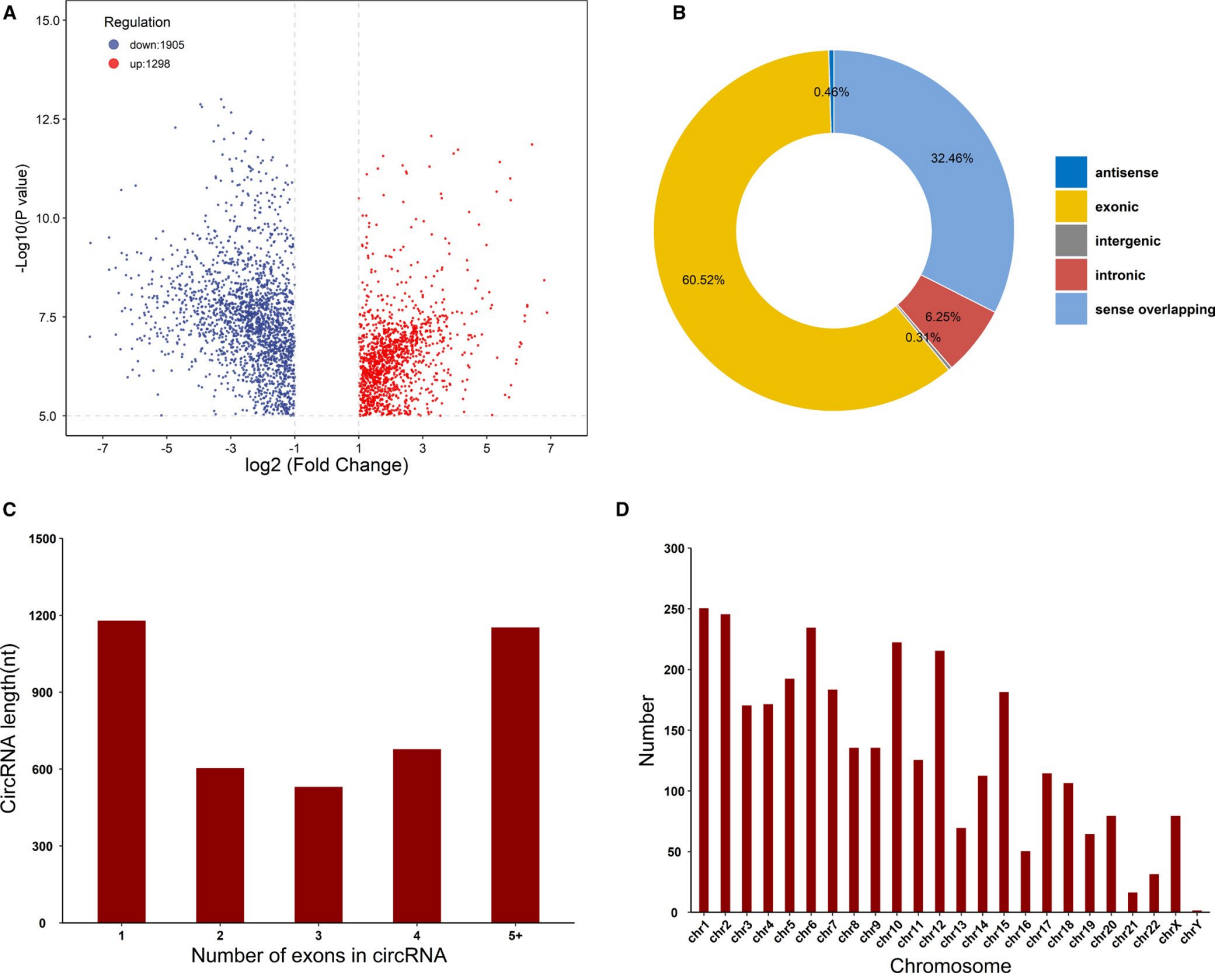
Proportion of differentially methylated N6‐methyladenosine regions. (A) Significant difference in the N6‐methyladenosine‐circRNA sites between the control and GBM groups (fold‐change ≥2 and *P* < .00001); (B) genomic distributions of N6‐methyladenosine‐circRNAs. The percentages of N6‐methyladenosine‐circRNAs measured under different conditions are presented in parentheses. (C) Length of circRNAs with different exon numbers in each gene. (D) Chromosomal distributions of differentially methylated regions in circRNAs

There were 1298 N6‐methyladenosine peaks distributed on 480 up‐methylated circRNAs and 1905 N6‐methyladenosine peaks distributed on 853 down‐methylated circRNAs. The top 10 down and up methylated N6‐methyladenosine sites in circRNAs are presented in Table 1. In addition, the N6‐methyladenosine‐circRNA sites were significantly differentially expressed between the GBM and control groups (fold‐change≥2.0, P≤0.00001; Figure 3A). The comparative analysis of differentially expressed N6‐methyladenosine‐circRNAs revealed that the most significant N6‐methyladenosine peaks were frequently composed of exonic sequences (Figure 3B). It has been reported that the majority of circRNAs are derived from protein‐coding genes (PCGs) that span 2‐3 exons. In this work, we found that most differentially methylated circRNAs originated from PCGs spanning a single exon, and the length of one exon in N6‐methyladenosine‐circRNAs was greater than that in two or more exons of N6‐methyladenosine‐circRNAs (Figure 3C). In addition, the distribution patterns of modified N6‐methyladenosine peaks in GBMs revealed that the abnormal N6‐methyladenosine peaks were ascribed to all chromosomes, but chromosomes 1, 2 and 6 were more prominently represented (Figure 3D). Among these chromosomes, the 3 chromosomes harbouring the highest number of differentially methylated N6‐methyladenosine peaks were chromosomes 1 (251), 2 (246) and 6 (235).

**TABLE 1:** Top 20 differently expressed N6‐methyladenosine peaks in GBMs in comparison with the controls

Chrom	PeakStart	PeakEnd	circRNA	Foldchange	Regulation
Chr12	46896701	46897080	Chr12:46870904‐46965195+	118.3	Up
Chr4	88619541	88619800	Chr4:88591257‐88631639−	111.2	Up
Chr18	2601141	2601760	Chr18:2585131‐2616530+	85.3	Up
Chr14	59993341	59993720	Chr14:59942587‐60018154−	76.9	Up
Chr1	224890341	224890680	Chr1:224868660‐224891733+	76.8	Up
Chr9	5 343 701	5344060	Chr9:5335468‐5361888−	73.0	Up
Chr6	6167690	6167851	Chr6:6167691‐6251162−	72.7	Up
Chr6	6144701	6144980	Chr6:6144613‐6145834−	67.7	Up
Chr9	14163361	14163680	Chr9:14120439‐14180865−	66.4	Up
Chr2	202187561	202187960	Chr2:202163961‐202228896−	65.1	Up
Chr6	62388791	62389140	Chr6:62388792‐62417308−	169.5	Down
Chr17	67144581	67145080	Chr17:67132233‐67148622−	167.4	Down
Chr5	175 533 061	175533132	Chr5:175501639‐175547983+	111.9	Down
Chr12	132837533	132837560	Chr12:132834228‐132839211−	111.7	Down
Chr6	62411881	62412120	Chr6:62388792‐62417308−	97.2	Down
Chr20	10279661	10279920	Chr20:10256077‐10286911+	96.9	Down
Chr12	121098221	121098440	Chr12:121097681‐121118298+	90.8	Down
Chr12	121104761	121105320	Chr12:121097681‐121118298+	87.5	Down
Chr8	1581181	1581212	Chr8:1574906‐1581212+	86.0	Down
Chr1	77512241	77512460	Chr1:77509889‐77534528+	85.0	Down

#### 3.2.3 Bioinformatic analyses of the altered N6‐methyladenosine circRNAs

**FIGURE 4:**
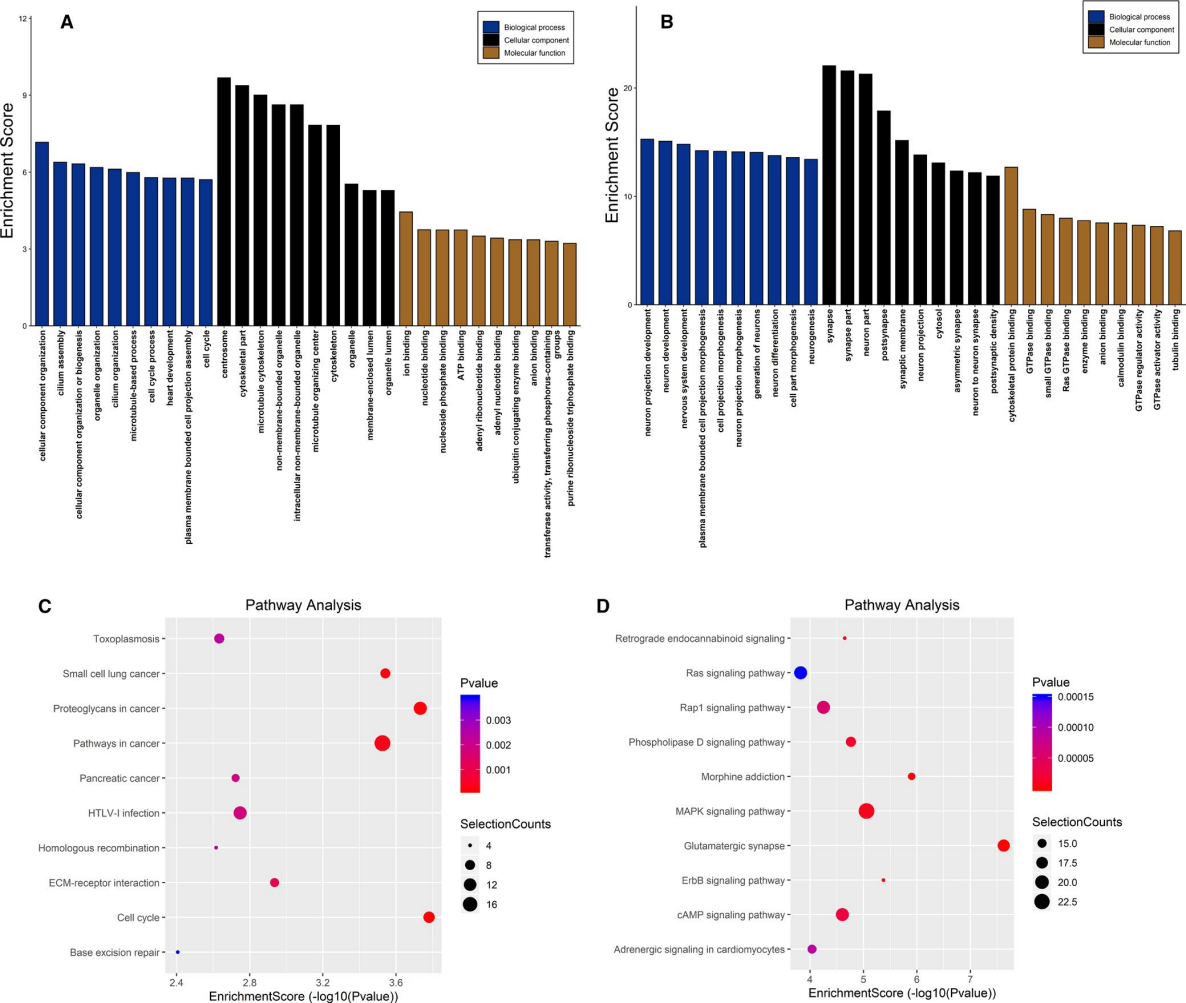
GO enrichment and KEGG pathway analyses of the N6‐methyladenosine peaks within circRNAs. (A, B) The most significant enrichment GO items of the up‐regulated and down‐regulated N6‐methyladenosine peaks within circRNAs; (C, D) the most significant enrichment pathways of the up‐regulated and down‐regulated N6‐methyladenosine peaks within circRNAs

To determine the pathophysiological role of N6‐methyladenosine modification in GBM, GO enrichment and KEGG pathway analyses were conducted on the modified N6‐methyladenosine peaks. As demonstrated in Figure 4A, the peaks that were up‐regulated in GBM were markedly associated with cellular component organization and cilium assembly (GO: biological process), centrosome and cytoskeletal part (GO: cellular component), and ion binding (GO: molecular function). Conversely, as presented in Figure 4B, the down‐regulated peaks were noticeably related to neuron projection development and neuron development (GO: biological process), synapse part (GO: cellular component) and cytoskeletal protein binding and GTPase binding (GO: molecular function). The KEGG results indicated that the up‐regulated peaks in GBM were remarkably associated with the cell cycle and proteoglycans in cancer (Figure 4C). In contrast, the down‐regulated peaks were obviously related to glutamatergic synapses and morphine addiction (Figure 4D).

### 3.3 Combined analysis of RNA sequencing and N6‐methyladenosine sequencing of circRNAs in the two sample groups

#### 3.3.1 circRNA sequencing and GO/KEGG pathway analysis

RNA sequencing identified 960 circRNAs overlapping in both the control and GBM groups, together with 4,482 and 2,146 circRNAs found only in the control group and GBM group, respectively (Figure S1A). A scatter plot was then employed to show the relationships of down and up regulated circRNAs (Figure S1B). Compared with the controls, 674 differentially expressed circRNAs (fold‐change ≥2.0, *P* ≤ .05) were identified in GBMs, including 454 up‐regulated and 220 down‐regulated circRNAs. Most of the circRNAs originating from PCGs spanned a single exon (Figure S1C), and the most differentially regulated circRNAs were composed of exonic sequences (Figure S1D). In addition, the distribution patterns of circRNAs in GBMs demonstrated that the altered circRNAs were attributed to all chromosomes, but chromosomes 1, 2 and 3 were overrepresented (Figure S1E).

In addition, the top 20 modified circRNAs are summarized in Table 2. GO and KEGG pathway analyses also highlighted that the top 10 functions were related to down or up regulated circRNAs (Figure S2A‐D). An upregulation trend was observed in most differentially expressed circRNAs.

**TABLE 2:** Top 20 differently expressed circRNAs in GBMs in comparison with the controls

Chrom	logFC	*P* Value	Regulation	Best transcript	Gene Name	Catalogue
Chr4:38091553‐38104778+	8.2964519	.0085734	Up	NM_015173	TBC1D1	Exonic
Chr3:145838899‐145842016−	8.2724149	.0087135	Up	NM_000935	PLOD2	Exonic
Chr2:29344240‐29358532+	8.1106292	.0097202	Up	NM_024692	CLIP4	Exonic
Chr17:43552466‐43553092−	8.075108	.0099558	Up	NM_014798	PLEKHM1	Exonic
ChrX:109507717‐109514082−	8.0053382	.010438	Up	NM_001025580	AMMECR1	Intronic
Chr10:27047991‐27059274−	7.99785	.0104883	Up	NM_005470	ABI1	Exonic
Chr7:129760589‐129762042+	7.9723325	.0106673	Up	NM_014997	KLHDC10	Exonic
Chr8:30938383‐30954366+	7.9558368	.0107845	Up	NM_000553	WRN	Exonic
Chr9:134814768‐134823218−	7.9204969	.0110457	Up	NM_004269	MED27	Sense overlapping
ChrX:24828015‐24861794+	7.9193002	.0110565	Up	NM_016937	POLA1	Exonic
Chr3:183368084‐183390272+	−9.9271413	.0090317	Down	NM_017644	KLHL24	Exonic
Chr3:183361268‐183390272+	−9.5725558	.0098728	Down	NM_017644	KLHL24	Exonic
Chr11:128993341‐129034322−	−9.4459541	.0101927	Down	NM_001142685	ARHGAP32	Exonic
Chr6:170846322‐170858201−	−9.1827746	.0108919	Down	NM_002793	PSMB1	Exonic
Chr7:16298015‐16317851−	−9.1170697	.0111323	Down	NM_001101417	ISPD	Exonic
Chr15:84228005‐84257523+	−8.9981061	.0117877	Down	NM_003027	SH3GL3	Exonic
Chr6:69723930‐69785930+	−8.7807487	.0131065	Down	NM_001704	ADGRB3	Exonic
Chr2:120885264‐120932580+	−8.6841013	.0137457	Down	NM_020909	EPB41 L5	Sense overlapping
Chr3:27420740‐27465643−	−8.427599	.0156709	Down	NM_001258379	SLC4A7	Exonic
Chr8:105080740‐105161076+	−8.4127533	.015797	Down	ENST00000408894	RIMS2	Exonic

#### 3.3.2 Correlation analysis between N6‐methyladenosine level and circRNA expression level

**FIGURE 5:**
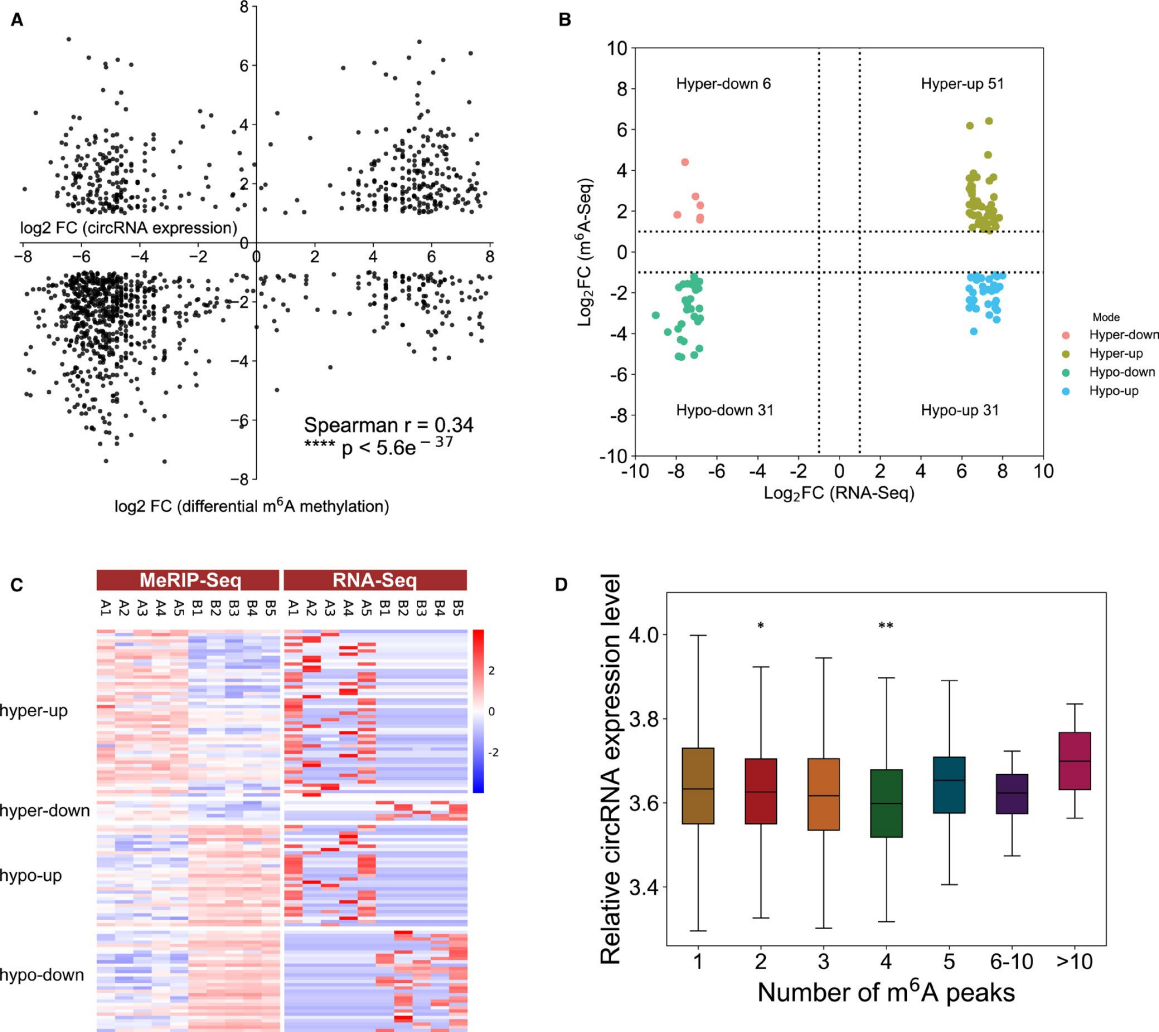
Cross‐analysis of N6‐methyladenosine‐RIP‐seq and RNA sequencing data. (A) Dot plot of Log2 FC (circRNA expression) against Log2 FC (differential N6‐methyladenosine methylation) indicates a significant correlation between total N6‐methyladenosine methylation and circRNA expression level (Spearman *r* = .34; *P* < 5.6e^−37^); (B) distributions of genes with remarkable changes in both N6‐methyladenosine and circRNA levels in GBM group samples compared with NC group samples (fold‐change >2, *P* < .05); (C) heat map of ‘hyper‐up’, ‘hyper‐down’, ‘hypo‐up’ and ‘hypo‐down’ genes represented in (B); (D) relative circRNA expression levels of transcripts harbouring different number of N6‐methyladenosine peaks. *P < .05 compared with the first column (N6‐methyladenosine peak = 1). GBM, Glioblastoma group; NC, Normal control group; FC, fold‐change

The N6‐methyladenosine sequencing data identified 3203 different methylated N6‐methyladenosine peaks in circRNAs, which were remarkably enriched (1298; hypermethylated) or suppressed (1905; hypomethylated) (fold‐change >2, *P* < .00001). Cross‐analysis of the N6‐methyladenosine sequencing and RNA sequencing data revealed a positive correlation of gene expression levels and differentially methylated N6‐methyladenosine peaks in GBM samples and NC samples (Spearman *r* = .34; Figure 5A). Among 57 hypermethylated N6‐methyladenosine sites detected by N6‐methyladenosine sequencing, we found 51 targets with up‐regulated circRNA expression (fold‐change >2, *P* < .05), referred to as ‘hyper‐up’. Six genes were found to have hypermethylated N6‐methyladenosine sites along with down‐regulated circRNA expression (fold‐change >2, *P* < .05), referred to as ‘hyper‐down’. In contrast, 31 of 62 genes with hypomethylated N6‐methyladenosine sites showed up‐regulated circRNA expression (fold‐change >2, *P* < .05), referred to as ‘hypo‐up’, and 31 of 62 genes with hypomethylated N6‐methyladenosine sites showed down‐regulated circRNA expression (fold‐change >2, *P* < .05), referred to as ‘hypo‐down’ (Figure 5B,C). Notably, the numbers of ‘hypo‐down’ and ‘hyper‐up’ genes were greater than those of ‘hypo‐up’ and ‘hyper‐down’ genes, and they also exhibited larger fold‐changes and smaller Ps (Figure 5B,C).

We wondered whether the number of N6‐methyladenosine peaks in each gene was related to gene expression levels. As shown in Figure 1E, different genes had different numbers of N6‐methyladenosine‐modified sites. By analysing the relative expression levels of these genes, it was observed that a smaller number of N6‐methyladenosine‐modified regions in each gene was related to elevated gene expression. Genes with two and four N6‐methyladenosine–modified sites tended to have less circRNA abundance than those with unique N6‐methyladenosine modification peaks (Figure 5D).

In addition, circRNAs with significant changed N6‐methyladenosine level and expression level (fold‐change >2, *P* < .05) were classified into the above four categories. To investigate the influence of the number of modification sites on the expression levels of different types of circRNAs, we have integrated the data between N6‐methyladenosine‐seq and RNA‐seq. The results showed that almost all circRNAs that met the requirements contained only one methyl assembly site, which indicates that the number of modification sites may not be directly related to the gene expression level. We collated and presented the intersection analysis table for the top 20 bits based on the fold change values (Tables [Link], [Link], [Link], [Link]). It also indicates that for a circRNA that only has one N6‐methyladenosine modification site, which is likely to be a potentially important molecule with significant changes in both methylation and expression levels. As gene expression is regulated by various factors, the impact of differential N6‐methyladenosine modifications on gene expression is worth further investigation.

#### 3.3.3 Effects of N6‐methyladenosine modification on circRNA expression in GBM

**FIGURE 6:**
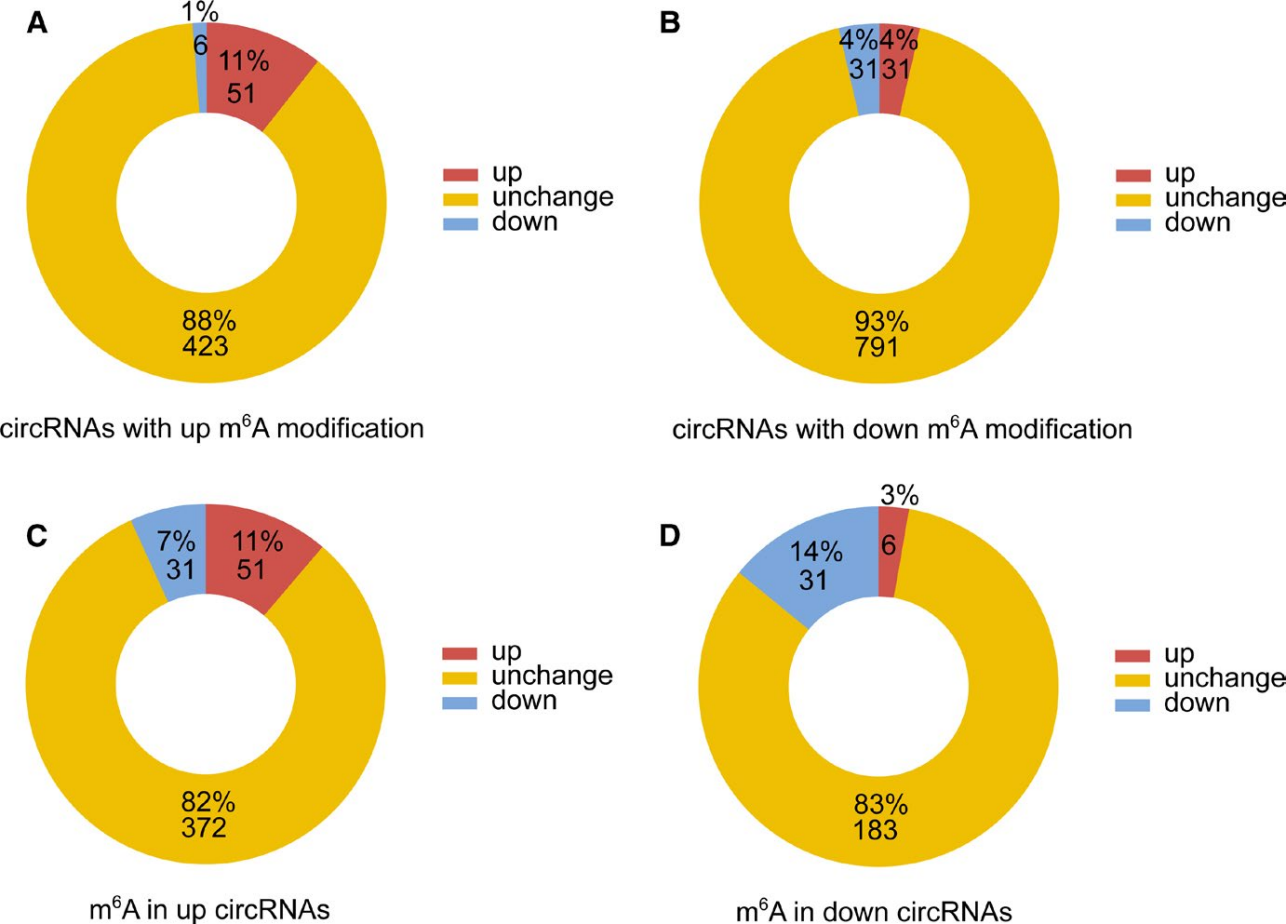
The association between N6‐methyladenosine modification and circRNA abundance in GBM. (A, B) The number and percentage of circRNAs were altered in GBM during N6‐methyladenosine modification. (C, D) The N6‐methyladenosine‐modified circRNAs were higher in the up‐regulated circRNAs than in the down‐regulated circRNAs. GBM, Glioblastoma group; NC, Normal control group

To determine whether N6‐methyladenosine methylation could affect circRNA expression levels, we examined the differential expression patterns of 2997 N6‐methyladenosine–modified circRNAs. Regardless of whether the N6‐methyladenosine modification of circRNAs was down‐regulated or up‐regulated, we observed that the levels of most circRNAs remained unchanged. However, we found that among the circRNAs with up‐regulated N6‐methyladenosine levels, more circRNAs exhibited increased expression levels than exhibited decreased expression levels (11.00% vs 6.00%) (Figure 6A,B). Among the genes with increased circRNA expression in the GBM group, more circRNAs showed increased N6‐methyladenosine levels than showed decreased N6‐methyladenosine levels (11.00% vs 7.00%). More N6‐methyladenosine circRNAs (18%) were detected among the up‐regulated circRNAs than among the down‐regulated circRNAs (17%) (Figure 6C,D). This result indicated that N6‐methyladenosine modification tends to exhibit a significant correlation with circRNA expression in GBM.

#### 3.3.4 Validation of molecules of interest (sites) based on MeRIP‐PCR

In combination with the RNA sequencing and N6‐methyladenosine sequencing data, we developed screening criteria: all circRNAs (including methylation sites) conforming to the criteria (fold‐change >2, *P* < .001) were ranked from high to low in terms of the size of fold‐change value, and then, the intersection of the two data was taken. CircRNAs with significantly higher levels of both methylation and expression (fold‐change >2, *P* < .001) in the GBM group were included in our further study. In conclusion, we selected five different circRNAs of interest (including five hypermethylated sites) according to the established screening criteria.

**FIGURE 7:**
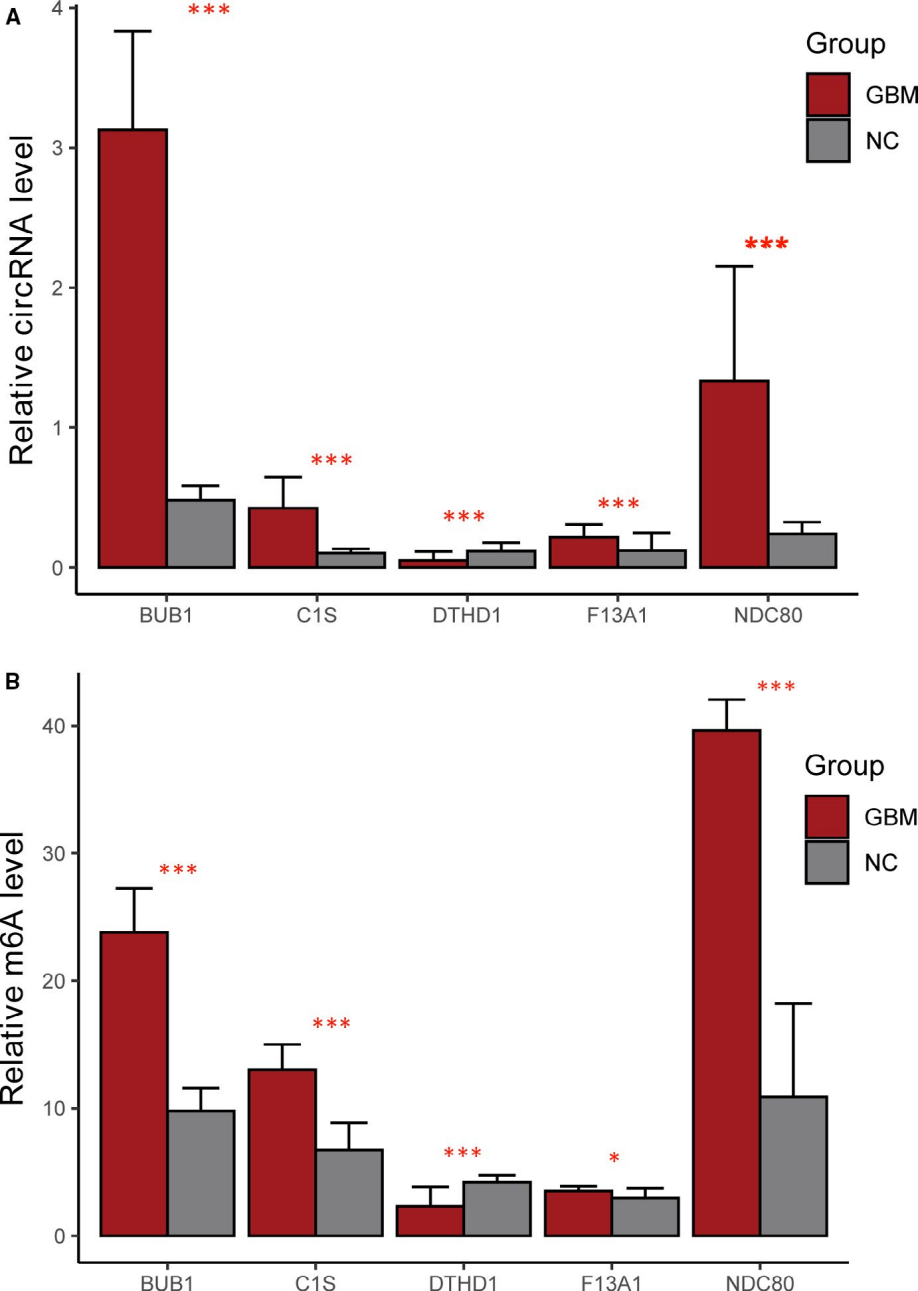
Gene‐specific N6‐methyladenosine qRT‐PCR assays and detection of global N6‐methyladenosine levels. (A) Relative circRNA levels of five representative genes were measured by real‐time PCR in NC group and GBM group samples; (B) gene‐specific N6‐methyladenosine qRT‐PCR verification of N6‐methyladenosine levels of 5 representative hypermethylated genes in NC group and GBM group samples. GBM: Glioblastoma group; NC: Normal control group

After the screening, to further confirm the accuracy of the N6‐methyladenosine sequencing data, we expanded the samples (10 samples in each group) to investigate the five target genes (BUB1, C1S, DTHD1, F13A1 and NDC80) that were used for gene‐specific N6‐methyladenosine qPCR analysis above. We observed that for four out of five genes, the N6‐methyladenosine level was consistent with the sequencing results, which proved the reliability of our transcriptome‐level N6‐methyladenosine sequencing data. Here, the inconsistency between the PCR validation results of circ‐DTHD1 and the results of sequencing (including data mining) may be related to heterozygous factors or interference in the sequencing process. In addition, the expression levels of the above circRNAs were detected in GBM and NC samples, and the results showed that the change trend of circRNA expression levels was similar to that of N6‐methyladenosine methylation levels (Figure 7A,B). In conclusion, GBM samples had unique N6‐methyladenosine modification patterns that are distinct from those of normal tissues at both the transcriptome‐wide and gene‐specific scales.

#### 3.3.5 Correlation analysis of the clinical prognosis of key molecules

Five genes (the mRNA form corresponding to circRNA) associated with up‐regulated N6‐methyladenosine levels in GBMs (BUB1, C1S, DTHD1, F13A1 and NDC80) were selected for further study. Among these, BUB1, C1S, DTHD1, F13A1 and NDC80 had elevated expression in the large cohort of GBM patients (n = 156) compared with normal controls (n = 5) (Figure S3A‐E). Different expression levels of C1S, DTHD1, F13A1 and NDC80 also had profound impacts on the overall survival rate of GBM patients. High C1S, DTHD1, F13A1 and NDC80 expression levels were associated with lower overall survival of GBM patients (Figure S3F‐J). In conclusion, based on TCGA data mining, we proved that the five key molecules screened by us are involved in the development of GBM at the mRNA level, and the circRNAs formed by their cyclization also have the characteristics of increased expression level and N6‐methyladenosine level in GBM, which can be used as the preferred molecules for later functional and mechanism studies.

In addition, although there is limited evidence at the gene database level to find direct associations between circRNAs and GBM prognosis, we identified several miRNAs that competitively bind circRNAs based on the molecular sponge function (ceRNA) of circRNAs and GBM disease association. And the data mining of GBM correlation was carried out. The specific screening process was as follows: There were about 2500 miRNAs in the miRbase, and about 600 miRNAs related to GBM in the miRCancer database. We selected 4 circRNAs with consistent sequencing and validation results. These 600 were used for circRNA‐miRNA analysis, and the top5 of each circRNA was selected to display for the results.

We performed patient‐based data analysis for each circRNA‐targeted binding miRNA and presented survival analysis for the four miRNAs most closely associated with prognosis (Figure S4A‐E). The results showed that these miRNAs were significantly positively correlated with the prognosis of glioma patients (*P* < .05), and played a potential role in promoting cancer. It is worth further study whether the specific mechanism of action is related to RNA methylation.

## 4 DISCUSSION

Many studies have suggested that epigenetic modification plays a crucial role in the pathogenesis of GBM.^20^, ^21^ Recently, N6‐methyladenosine modification has attracted extensive attention, but the specific mechanism of this novel RNA modification in the occurrence and development of GBM has not been fully studied, especially in circRNA.^11^ We used RNA N6‐methyladenosine sequencing to explore the state of N6‐methyladenosine‐circRNA modifications in GBM tissues. The results fully demonstrated that the N6‐methyladenosine modification status of circRNAs in the GBM group was noticeably different from that in the NC group, and these differences are likely to participate in the regulation of tumorigenesis and biological behaviour.

Next, we addressed the importance of N6‐methyladenosine labels on the exons that produce circRNAs. A recent study reported that among all circRNAs in human embryonic stem cells, N6‐methyladenosine‐circRNAs are generally encoded by single exons and are longer than those encoded by multiple exons.^12^ Interestingly, we found the same pattern in GBM tissues. In addition, studies have shown that the N6‐methyladenosine regions are most commonly located in the last exon, but the cyclization of the last exon of the gene is not common, so it is speculated that different sets of rules may govern the application of N6‐methyladenosine in circRNAs and mRNAs.^22^, ^23^

Our results showed that a total of 3,203 N6‐methyladenosine peaks in circRNAs were significantly differentially expressed between the two groups (fold‐change >2, *P* < .00001), of which 1,298 were up‐regulated and 1,905 were down‐regulated. RNA sequencing showed that 454 up‐regulated and 220 down‐regulated circRNAs were detected in the GBM group (fold‐change >2, *P* < .05). In addition, through the combined analysis of N6‐methyladenosine modification levels and circRNA expression levels, we observed that the level of N6‐methyladenosine at circRNA (sites) increased, with the exception of circRNA expression level does not change, also increase the expression level of circRNA in most of the other, which means that circRNA expression level was positively related to the level of N6‐methyladenosine modification, and it can be speculated that the assemblies at N6‐methyladenosine sites promote circRNA expression. These data highlight the dynamic characteristics and intrinsic relationship between N6‐methyladenosine modification in circRNAs and N6‐methyladenosine circRNA expression in GBM.

The N6‐methyladenosine modification process of circRNA is similar to that of mRNA in that it is mainly carried out by methyltransferase complexes consisting of METTL3, METTL14, WTAP and other components, which can be reversed by demethylases (eg FTO and ALKBH5).^24^, ^25^ The content of total RNA N6‐methyladenosine in GBM is increased, and the specific influence of N6‐methyladenosine modification on gene expression depends to a large extent on the function of downstream N6‐methyladenosine readers, which are associated with the multiple effects of N6‐methyladenosine on gene expression and affect the stability, localization, splicing, nuclear output and translation.^26^, ^27^, ^28^, ^29^ Normally, N6‐methyladenosine modification can regulate mRNA stability and is mediated by YTHDF2, but by contrast, there does not appear to be a similar mechanism for promoting circRNA degradation as there is for mRNA. In addition, N6‐methyladenosine‐circRNAs can further modify their capability to interact with YTH and other RNA‐binding proteins.^26^, ^30^, ^31^, ^32^ Zhang et al found that circRNAs derived from SHPRH can encode tumour suppressor proteins.^33^ In addition, SMO‐193a.a is encoded by circRNA and drives the genesis and progression of GBM.^34^ Recent studies have shown that N6‐methyladenosine modifications mediate the generation of translatable circRNAs, which provides an important research basis and direction for further research on the modifications and new functions of circRNAs.^35^

Of the five circRNAs of interest we selected, BUB1 has been considered a novel therapeutic target for glioblastoma and plays a key role in promoting tumour proliferation and radiation resistance in GBM in a forkhead box protein M1 (FOXM1)–dependent manner.^36^ In addition, ZWINT is significantly positively correlated with the mitochondrial protein NDC80, the serine/threonine protein kinase PLK1 (PLK1), and complex spindle and mitochondrial‐related subunit 1 (SKA1) and is associated with the regulation of mitosis and the cell cycle in GBM.^37^ In primary glioblastoma, the low tumour copy number of the F13A1 gene fragment is associated with poor survival,^38^ which is inconsistent with our sequencing results; this complex mechanism needs further study. These key molecules can be used as the preferred genes for future research on the function and mechanism of N6‐methyladenosine–mediated GBM development.

This study also has some limitations. Further cellular functional experiments and in vivo experiments are needed to confirm the regulatory effect of N6‐methyladenosine RNA modification on GBM gene expression and to determine the correlation between N6‐methyladenosine modification and the origin and progression of GBM. In addition, the knockout or overexpression of key enzymes involved in N6‐methyladenosine modification may also be a good strategy and emerging direction for the study of N6‐methyladenosine methylation–mediated tumour cell responses.^39^, ^40^, ^41^, ^42^

## 5 CONCLUSION

Our findings provide the first map of human circRNA N6‐methyladenosine modification in GBM and identify differentially expressed circRNA transcripts associated with hypermethylated and hypomethylated modifications, revealing the potential association between abnormal N6‐methyladenosine modifications and cancer‐related gene expression and function, which is helpful to further study the mechanism of N6‐methyladenosine–mediated gene expression regulation. We hope that it can provide a road map for discovering the mechanism of action of N6‐methyladenosine modification in noncoding RNAs and the development of new therapeutic strategies for GBM or provide new ideas for further research by regulating N6‐methyladenosine modification transcripts or N6‐methyladenosine–related genes.

## CONFLICT OF INTEREST

The authors declare that they have no conflicts of interest.

## AUTHOR CONTRIBUTIONS

**Yuhao Zhang:** Conceptualization (equal); Writing‐original draft (equal). **Xiuchao Geng:** Conceptualization (equal); Writing‐original draft (equal). **Jianglong Xu:** Writing‐original draft (equal). **Qiang Li:** Methodology (equal). **Liangchao Hao:** Methodology (equal). **Zhaomu Zeng:** Investigation (supporting); Software (supporting); Validation (supporting). **Menglin Xiao:** Investigation (supporting); Software (supporting); Validation (supporting). **Jia Song:** Investigation (supporting); Validation (supporting). **Fulin Liu:** Project administration (equal); Writing‐review & editing (equal). **Chuan Fang:** Project administration (equal); Writing‐review & editing (equal). **Hong Wang:** Data curation (equal); Project administration (equal); Supervision (equal); Writing‐review & editing (equal).

## Supporting information

Fig S1‐S4

Tab S1

Tab S2

Tab S3

Tab S4

Tab S5

Tab S6

## Data Availability

The data sets used and analysed during the present study are available from the corresponding author on reasonable request.
