# Supplementary material for: Identification and characterization of N6‐methyladenosine modification of circRNAs in glioblastoma
Source: J Cell Mol Med. 2021 Jun 27;25(15):7204–17. doi: 10.1111/jcmm.16750 (PMC8335669; doi:10.1111/jcmm.16750)
Supplement: Supplementary file 1 — Fig S1‐S4 [file JCMM-25-7204-s002.pdf]

Supplementary Figures

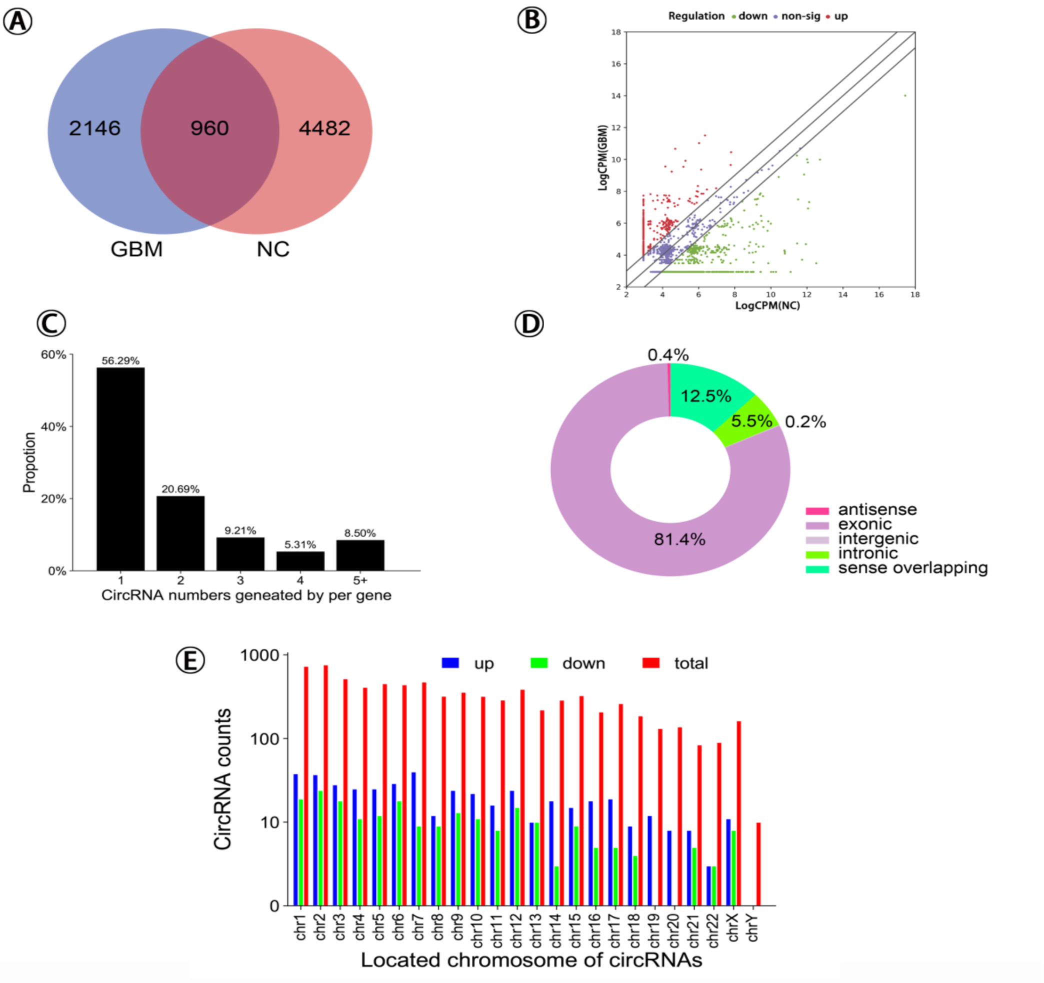

Figure S1. CircRNA profiling in control subjects versus GBM patients. (A) Comparison of the expression circRNAs identified in control subjects and GBM patients by Venn diagram; (B) Scatter plots showing that the circRNAs were significantly differentially expressed between control subjects and GBM patients (fold-change ⩾2.0 and P<0.05); (C) Proportion of circRNAs harbouring different numbers per gene; (D) Genomic distributions of N6-methyladenosine-circRNAs. The percentages of N6-methyladenosine-circRNAs measured under different conditions are presented in parentheses; (E) Chromosomal distributions of the differentially expressed circRNAs. GBM: Glioblastoma group; NC: Normal control group.

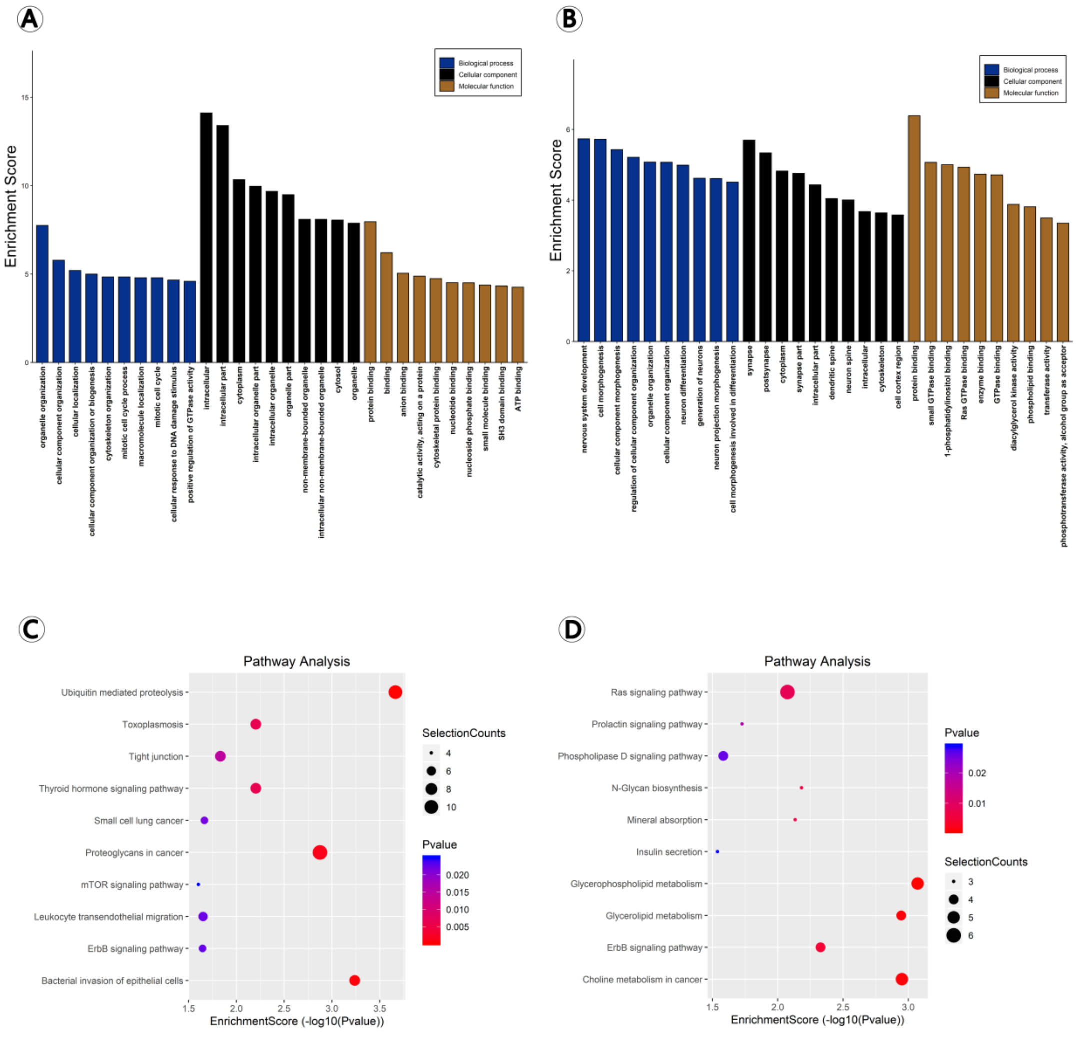

Figure S2. GO enrichment and KEGG pathway analyses for circRNAs. (A, B) The most significant enrichment GO items of the upregulated and downregulated circRNAs; (C, D) The most significant enrichment pathways of the upregulated and downregulated circRNAs.

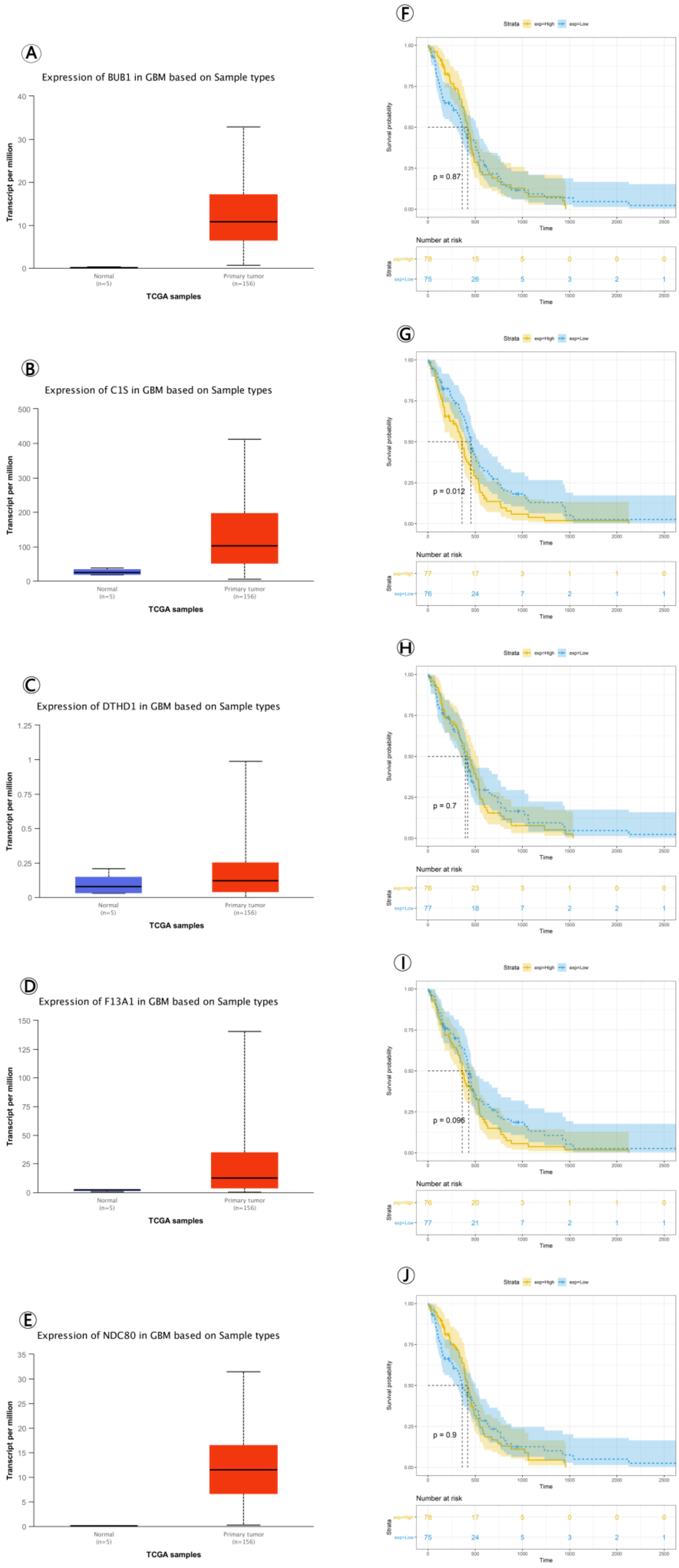
A
Expression of BUB1 in GBM based on Sample types
B
Expression of C1S in GBM based on Sample types
C
Expression of DTHD1 in GBM based on Sample types
D
Expression of F13A1 in GBM based on Sample types
E
Expression of NDC80 in GBM based on Sample types
Transcript per million
Normal (n=5)
Primary tumor (n=156)
TCGA samples
F
p = 0.87
G
p = 0.012
H
p = 0.7
I
p = 0.096
J
p = 0.9
Strata exp=High exp=Low
Survival probability
Time
Number at risk

Figure S3. The relationship of BUB1, C1S, DTHD1, F13A1 and NDC80 expression with GBM patients in a large cohort. (A-E) Expression of BUB1, C1S, DTHD1, F13A1 and NDC80 in a large cohort of GBM patients (n=156) compared with normal controls (n=5); (F-J) Overall survival of GBM patients with different expression levels of BUB1, C1S, DTHD1, F13A1 and NDC80. Data were analysed by the UALCAN online analysis tool based on TCGA.

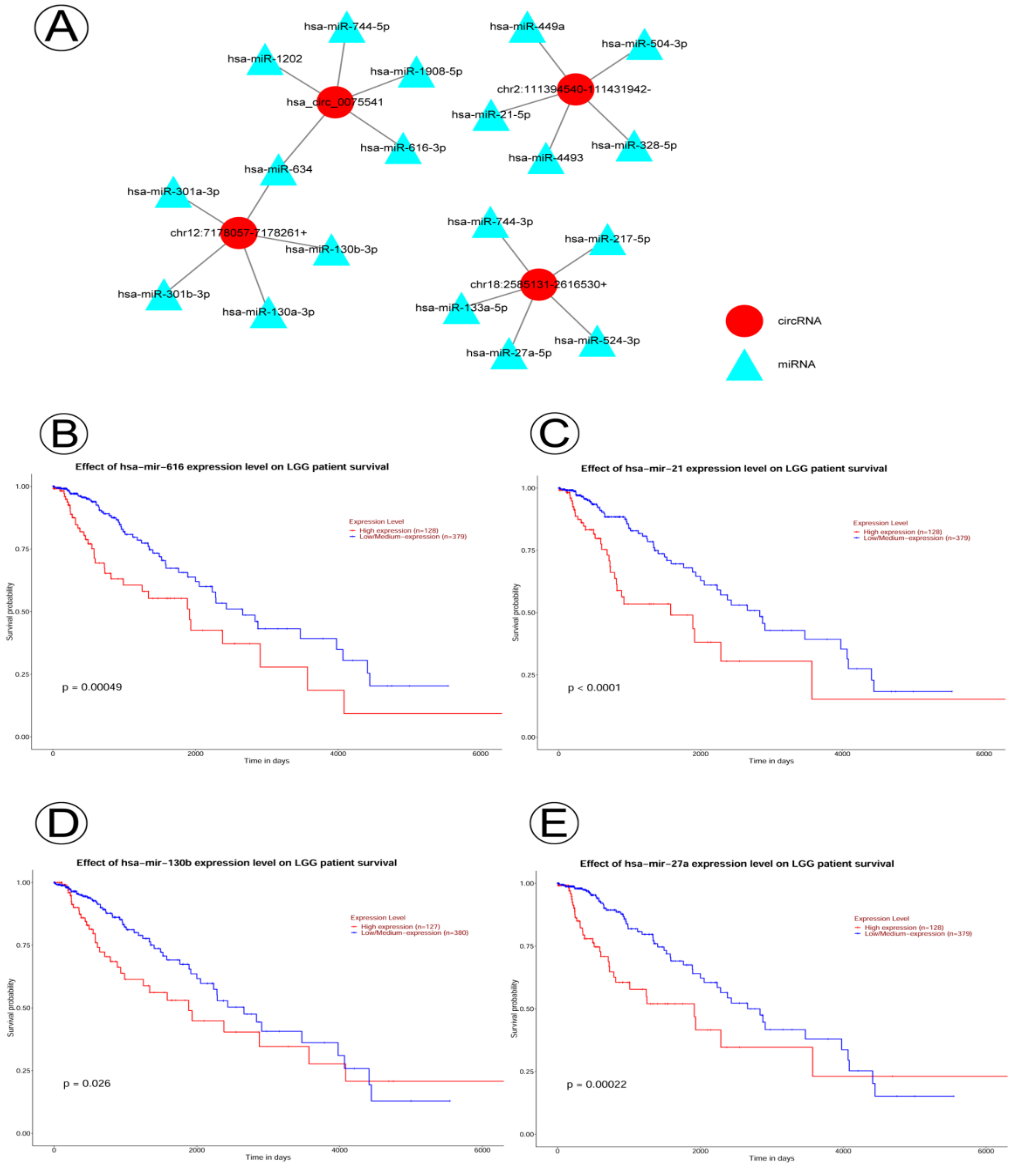

Figure S4. CircRNA-miRNA interaction analysis and miRNA survival analysis.

(A) CircRNA-miRNA interaction analysis (Top 5); (B-E) Overall survival of glioma patients with different expression levels of hsa-miR-616/21/130b/27a. Data were analysed by the UALCAN online analysis tool based on TCGA.
