# Supplementary material for: Identification and characterization of N6‐methyladenosine modification of circRNAs in glioblastoma
Source: J Cell Mol Med. 2021 Jun 27;25(15):7204–17. doi: 10.1111/jcmm.16750 (PMC8335669; doi:10.1111/jcmm.16750)
Supplement: Supplementary file 2 — Tab S1 [file JCMM-25-7204-s004.docx]

**Table S1. Clinical information for relevant patients in this study**

| Patient | Age(y) | Gender | WHO grade |
| --- | --- | --- | --- |
| GBM1 | 62 | Male | WHO IV |
| GBM2 | 67 | Female | WHO IV |
| GBM3 | 52 | Male | WHO IV |
| GBM4 | 63 | Female | WHO IV |
| GBM5 | 78 | Female | WHO IV |
| NC1 | 45 | Female | - |
| NC2 | 51 | Male | - |
| NC3 | 65 | Female | - |
| NC4 | 41 | Male | - |
| NC5 | 55 | Male | - |
