# Supplementary material for: Identification and characterization of N6‐methyladenosine modification of circRNAs in glioblastoma
Source: J Cell Mol Med. 2021 Jun 27;25(15):7204–17. doi: 10.1111/jcmm.16750 (PMC8335669; doi:10.1111/jcmm.16750)
Supplement: Supplementary file 3 — Tab S2 [file JCMM-25-7204-s003.docx]

**Table S2. Sequences of primers used for qRT-PCR of circRNA N6-methyladenosine levels**

| **Gene name** | **Sequence** |
| --- | --- |
| BUB1 | F: 5 CAGAGGCAATCACACCCCAT 3 |
|  | R: 5 AGGGTCATTGCCCTTGTAGC 3 |
| C1S | F: 5 GGGCACTCCTTTCTTGCACT 3 |
|  | R: 5 CCTCTAGCAAGGCGTCTCAA 3 |
| DTHD1 | F: 5 GCACCACTGCCTGCATAGTA 3 |
|  | R: 5 TTGGGGGCCAGTAACATCAC 3 |
| F13A1 | F: 5 CTGGCCAAGCAAAAGTCCAC 3 |
|  | R: 5 ACAGGCAAGTGTCCAGGATG 3 |
| NDC80 | F: 5 TGGTTGCTACACATGTTGGGT 3 |
|  | R:5 TTTCTTCTCCCCAAGGCTGC 3 |
